# Effects of Olive Pomace Powder Incorporation on Physicochemical, Textural, and Rheological Properties of Sheep Milk Yogurt

**DOI:** 10.3390/foods14173118

**Published:** 2025-09-06

**Authors:** Angela Carboni, Roberto Cabizza, Pietro Paolo Urgeghe, Francesco Fancello, Severino Zara, Alessandra Del Caro

**Affiliations:** Dipartimento di Agraria, Università degli Studi di Sassari, Viale Italia 39, 07100 Sassari, Italy; a.carboni42@studenti.uniss.it (A.C.); paolou@uniss.it (P.P.U.); fancello@uniss.it (F.F.); szara@uniss.it (S.Z.); delcaro@uniss.it (A.D.C.)

**Keywords:** by-product, fortification, functional food, olive pomace, sheep milk yogurt

## Abstract

The valorization of agro-industrial by-products is a key component of sustainability goals in food production. Olive pomace (OP), a major by-product of olive oil extraction, is characterized by a high content of dietary fiber and bioactive phenolic compounds with antioxidant activity, which contribute to its nutritional and functional potential. The present study investigated the effect of the fortification of sheep milk yogurt with freeze-dried OP (1% *w*/*w*), added either before pasteurization (YOPB) or after overnight refrigeration (YOPA). The OP showed considerable antioxidant capacity and was microbiologically safe. Its addition significantly increased the yogurt’s total phenolic content, with YOPB displaying the lowest syneresis and the highest water-holding capacity and apparent viscosity. Textural and scanning electron microscopy analyses revealed that the timing of OP addition affected the gel structure, with pre-pasteurization incorporation facilitating a superior integration into the protein network. The microbial viability was preserved, and the sensory evaluation showed no significant differences in consumer acceptance between the control and YOPB. These findings highlight OP’s potential as a functional ingredient for dairy fortification, contributing to waste reduction and improving gel structure. The findings obtained provide support for the development of sustainable and functional dairy products enriched with by-products derived from the olive oil industry.

## 1. Introduction

In recent years, the valorization of agri-food by-products has emerged as a strategic priority, driven by the dual imperative of reducing environmental impact and enhancing the sustainability of food production systems. The accumulation of large quantities of food processing residues represents not only an environmental concern but also a significant economic loss [1]. This has led to increasing scientific interest in the recovery and reuse of bioactive compounds from such by-products, with the aim of reintroducing them into the food chain as functional ingredients or value-added components [2]. Such practices contribute to the development of a circular and sustainable food economy [3,4,5].

Among the plethora of by-products, olive pomace (OP), the primary solid residue generated during the extraction of extra virgin olive oil from *Olea europaea* L., has received increasing attention. Olive oil production is a key agro-industrial activity in the Mediterranean region, and it generates substantial amounts of OP [6,7]. Due to its high organic load and phytotoxic potential, OP is often regarded as an environmental burden [8]. Nevertheless, its composition, which is rich in phenolic compounds originally present in olives and which have been widely studied for their involvement in key biological processes, dietary fiber, and residual oil, has highlighted its potential as a source of functional ingredients for food applications and sustainable valorization strategies [9]. Among these compounds, OP contains a wide range of bioactive molecules, including hydroxytyrosol, tyrosol derivatives, iridoid precursors, flavonoids, lignans, phenolic acids, secoiridoids, and related compounds, all of which have been associated with beneficial health effects [10]. These include antioxidant, anti-inflammatory, and antimicrobial properties, supporting OP’s potential use in nutraceuticals and functional foods such as beverages, pasta, and bakery products [11,12,13,14,15].

Sheep milk is considered an excellent matrix for functional enrichment due to its high protein content, favorable lipid profile, and richness in bioactive peptides and micronutrients [16,17,18]. Moreover, sheep dairy production holds significant economic and cultural relevance in several Mediterranean countries [19], particularly in Greece (943,970 t), Spain (529,900 t), and Italy (471,290 t) [20]. In Italy, the island of Sardinia alone accounts for approximately 68.1% of the national sheep milk output [21].

Concurrently, there has been growing interest in the development of value-added fermented dairy products, such as probiotic yogurts and functional beverages [22,23]. Building on this evidence, recent studies have examined the incorporation of olive pomace into fermented dairy products, particularly cow milk yogurts, as a strategy to enhance their nutritional and functional profiles while promoting the sustainable reuse of agro-industrial by-products [24,25]. Overall, studies incorporating OP into cow’s milk yogurts indicated enhancements in phenolic content and antioxidant activity, along with the potential for promoting health. These studies have not reported any significant adverse effects on product quality or consumer acceptance [24,25]. However, to date, no studies have examined its incorporation into sheep milk for yogurt production.

This study aims to develop novel functional yogurts by fortifying sheep milk with olive pomace derived from the Bosana cultivar, which is typical of Sardinia, added either before pasteurization or after the refrigeration process. The resulting yogurts were subsequently evaluated in terms of their physicochemical, microbiological, rheological, structural, and sensory properties.

## 2. Materials and Methods

### 2.1. Sampling and Preparation of OP

OP was collected from a Sardinian olive oil mill (Accademia Olearia Srl, Alghero, Italy). The olives (Bosana cv., an autochthonous Sardinian cultivar) were harvested during the 2021–2022 season in the Alghero area (latitude 40°33′ N; longitude 8°18′ E, Sardinia, Italy). The freshly obtained OP was immediately frozen and stored at −20 °C and then freeze-dried using a Labconco 8L −50 °C series freeze-dryer (Kansas City, MO, USA) at a collecting temperature of −54.0 °C and a pressure of 0.1 mbar. The dried material was ground into a fine powder using an ultra-centrifugal mill (ZM 200, Retsch GmbH, Haan, Germany), vacuum-packed (VAC VM 151 HG, Audion Elektro B.V., Weesp, The Netherlands), and stored in the dark at room temperature until further analysis and use as a fortification ingredient.

### 2.2. Proximate Composition, Water Activity, and Total Dietary Fiber of OP

The proximate composition of OP was determined using standardized gravimetric and instrumental methods. Moisture content was assessed by weighing 3 g samples in ceramic crucibles and drying them in an oven at 105 °C until constant weight was reached [26]. Ash content was measured by incineration in a muffle furnace at 550 °C, following the AOAC 923.03 official method [27]. Lipid content was determined in accordance with the AOAC 2003.06–2006 method [28] using a solvent extraction system (SER 158, Velp Scientific, Deer Park, NY, USA) and petroleum ether (Carlo Erba Reagents, Milan, Italy) as the solvent. The results were measured gravimetrically.

Protein content (%) was estimated by the quantification of carbon (C), hydrogen (H), and nitrogen (N) in OP, following the method described by Dahdah et al. [6]. This was achieved using a LECO 628 elemental analyzer (LECO Corporation, St. Joseph, MI, USA), calibrated in accordance with the AOAC Official Method 990.03 [29]. The nitrogen values, expressed as % (*w*/*w*), were converted to crude protein by applying the conventional factor of 6.25.

Digestible carbohydrates were then estimated by the difference, subtracting from 100 the percentages of protein, lipids, total dietary fiber, and ash.

Water activity (a_w_) was measured using an electronic hygrometer (Aw-Win, Rotronic) equipped with a Karl-Fastprobe, calibrated with standard LiCl solutions over a range of 0.10–0.95 a_w_ [30].

Total dietary fiber (TDF) was quantified gravimetrically using a K-TDFR enzymatic kit (Megazyme International Ireland Ltd., Bray, Ireland), in accordance with the official methods [31,32]. The filtration step was performed with a CSF6 filtration unit (VELP Scientifica Srl, Usmate, Italy).

### 2.3. Total Phenolic Content of OP

The extraction of total phenolic content (TPC) from OP was carried out according to the procedure described by Cannas et al. [33]. Briefly, the ultrasound-assisted extraction (UAE) procedure was performed in an ultrasonic bath (ARGO Lab, model DU-100, Carpi, Italy) at a constant frequency of 40 kHz and a power of 144 W for 10 min at 38 °C. The extraction medium contained 1 g of OP and 20 mL of a mixture of ethanol and water (40:60, *v*/*v*). Following sonication, the mixture was centrifuged at 5700 rpm for 10 min at 4 °C (SL1R Plus, Thermo Scientific, Waltham, MA, USA). The resulting supernatant was collected and filtered through a 0.45 µm cellulose acetate membrane (Artiglass, Due Carrare, Italy).

TPC was determined using the Folin–Ciocalteu colorimetric method as described by Noriega-Rodríguez [34], with slight modifications. Briefly, 1 mL of extract was mixed with 7.5 mL of water and 0.5 mL of Folin–Ciocalteu reagent. The solution was vortexed and incubated in the dark for 3 min. Subsequently, 1 mL of 10% (*w*/*v*) Na_2_CO_3_ solution was added, and the mixture was vortexed (ZX3 Velp Scientifica, Usmate, Italy) again and kept in the dark at room temperature for 90 min. Absorbance was measured at 765 nm using a UV–VIS spectrophotometer (Cary 3500, Agilent, Cernusco, Milan, Italy). A calibration curve was constructed using gallic acid (Sigma-Aldrich, Milan, Italy) at concentrations ranging from 0.01 to 1 mg/mL. TPC values were expressed as g of gallic acid equivalent (GAE) per kg of dry weight (g GAE/kg DW).

### 2.4. Antioxidant Activity of OP

The antioxidant activity (AA) of OP was assessed on organic extracts obtained according to the method described by Collar et al. [35] with some modifications. Briefly, 2 g samples of olive pomace were extracted with 20 mL of acidified methanol/water (MeOH/H_2_O 50:50 *v*/*v*, pH 2; Carlo Erba, Milan, Italy) under stirring at room temperature for 60 min. The mixture was centrifuged at 4800 rpm for 10 min (SL1R Plus, Thermo Scientific, Waltham, MA, USA), and the resulting supernatant was recovered. The residue from the first extraction was then subjected to a second extraction with 70% (*v*/*v*) acetone solution (Carlo Erba, Milan, Italy), followed by centrifugation under the same conditions. Subsequently, the two supernatants were combined, and the volume was adjusted to 25 mL with methanol.

Antioxidant activity was assessed by an ABTS assay, following the procedure of Dahdah et al. [7], and the results were expressed as µmol of Trolox equivalent per gram of dry weight (µmol TE/g DW). The ABTS•^+^ radical cation was generated by reacting 7.4 mM ABTS (Sigma-Aldrich, Milan, Italy) with 2.6 mM potassium persulfate (Sigma-Aldrich, Milan, Italy) in reverse-osmosis water. The solution was kept in the dark at room temperature for 12 h to allow for complete radical formation. On the following day, the stock solution was diluted with phosphate-buffered saline (PBS, 0.1 M, pH 7.4) to achieve an absorbance of 0.70 ± 0.02 at 734 nm, measured using a UV–VIS spectrophotometer (Cary 3500, Agilent Technologies, Cernusco, Milan, Italy). All measurements were performed in triplicate. Antioxidant activity was quantified using a standard curve prepared with Trolox (Sigma-Aldrich, Milan, Italy).

### 2.5. Color of OP

The color attributes of OP were measured using a colorimeter equipped with a CR-300 measuring head (Minolta CR-300, Konica Minolta Sensing, Ramsey, NJ, USA), according to the CIE Lab color space system (L*, a*, b*), using D65 as the standard illuminant and a 10° observer angle. The instrument was calibrated prior to analysis using the white calibration tile provided by the manufacturer.

### 2.6. Scanning Electron Microscopy Analysis of OP

The olive pomace powder microstructure was examined using scanning electron microscopy (SEM). Samples were initially fixed in 2.5% glutaraldehyde (Sigma-Aldrich, Merck KGaA, Darmstadt, Germany) in 0.1 M phosphate buffer (pH 7.2) for 24 h and then post-fixed in 1% osmium tetroxide (Merck Life Science Srl, Milan, Italy) for 1 h. Subsequently, samples were dehydrated through a graded ethanol series, dried using critical point drying (CPD) in a CO_2_ chamber (Jumbo, SPI Supplies, West Chester, PA, USA), and coated with a gold/palladium layer using a sputter coater (S150A, Edwards High Vacuum International, Crawley, UK) to ensure conductivity. SEM analysis was carried out using a Zeiss EVO LS10 microscope (Carl Zeiss Microscopy GmbH, Oberkochen, Germany) operated in high-vacuum mode. The particle size distribution was expressed as equivalent circular diameter (ECD). This was calculated from the particle areas measured on SEM micrographs. The ImageJ software (version 1.51n, Wayne Rasband, National Institutes of Health, Bethesda, MD, USA) was utilized for this purpose. The data were reported as mean values, ranges, and percentage distributions across predefined size classes (<1 µm; 1–5 µm; 5–10 µm; 10–50 µm; >50 µm).

### 2.7. Microbiological Analysis of OP

The freeze-dried OP powder was subjected to microbiological analysis to assess the presence of spoilage microorganisms and potential pathogens. Detection of *Listeria monocytogenes* and *Salmonella* spp. was carried out following ISO 11290-1/2 and ISO 6579-1 [36,37,38], respectively.

*Pseudomonas* spp. was enumerated on Pseudomonas Agar Base (Neogen, Lansing, MI, USA) supplemented with 2 vials of CFC Supplement (Cetrimide 5 mg/L, Fusidic Acid 5 mg/L Cephalothin 25 mg/L, Neogen, Lansing, USA). The contents of each vial were reconstituted by adding 5 mL of sterile 50% ethanol. Plates were incubated at 30 °C for 48 h [39]. *Enterobacteriaceae* were counted on MacConkey Agar (VWR, Milan, Italy) after incubation at 37 °C for 24 h [40]. *Escherichia coli* was enumerated on TBX (tryptone bile X-glucuronide, VWR, Milan, Italy) Agar at 44 °C. Presumptive enterococci were enumerated on bile esculin agar (Neogen, Lansing, MI, USA) and incubated at 37 °C for 48 h [41].

Coagulase-positive staphylococci were counted using Baird–Parker Agar (Oxoid, Basingstoke, UK) supplemented with egg yolk tellurite emulsion (Oxoid, Basingstoke, UK), followed by incubation at 37 °C for 24 h [42]. Typical colonies were confirmed using the Oxoid Staphylase Test (Oxoid, Basingstoke, UK). Aerobic spore-forming bacteria were enumerated on Tryptic Soy Agar (VWR, Milan, Italy) plates inoculated with heat-treated dilutions (80 °C for 10 min) and incubated at 30 °C for 72 h [43].

Yeasts and moulds were enumerated on Rose Bengal Chloramphenicol Agar (VWR, Milan, Italy) and incubated at 25 °C for 48 h [44].

### 2.8. Yogurt Production

#### 2.8.1. Raw Sheep Milk Analysis

The sheep milk employed in the production of yogurt was sourced from a local farm in Sassari, Italy, and obtained from healthy Sarda breed ewes that had not been treated with antibiotics. Milk composition was analyzed using a Milkoscan FT+ (Foss Electric, Hillerød, Denmark). The parameters evaluated included fat, protein, casein, lactose, and urea.

The mean chemical composition of the milk was as follows: pH 6.77 ± 0.02; fat 5.55 ± 0.28 (%, *w*/*w*); protein 4.69 ± 0.10 (%, *w*/*w*); fat/protein ratio 1.18 ± 0.03; casein 3.46 ± 0.13 (%, *w*/*w*); lactose 4.63 ± 0.10 (%, *w*/*w*); urea 0.03 ± 0.01 (%, *w*/*w*).

The somatic cell count (SCC) was determined separately using a Fossomatic 5000 (Foss Electric, Hillerød, Denmark), with a mean value of 1344 ± 530 × 10^3^ cells/mL.

#### 2.8.2. Lab-Scale Yogurt-Making Process

**Figure 1:**
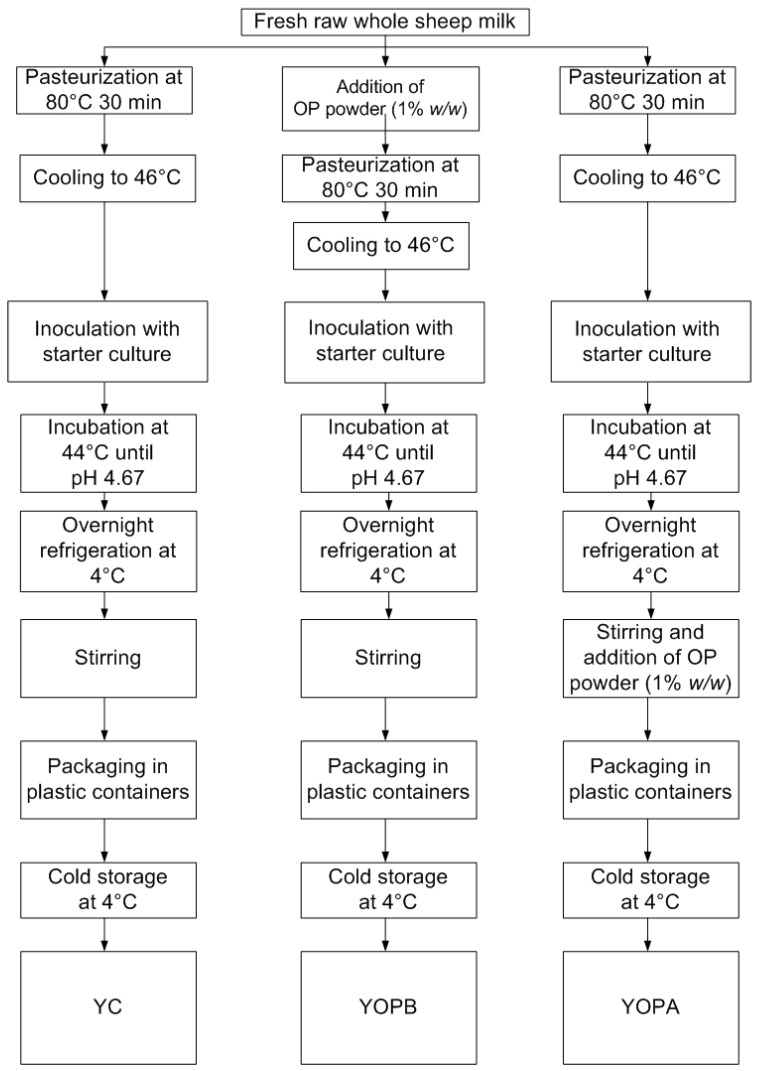
Experimental workflow for the production of YC, YOPB, and YOPA sheep milk yogurt.

All yogurt formulations were repeated in triplicate within a short timeframe to ensure consistency in the raw milk composition and to avoid potential batch-to-batch variations. Bulk raw whole sheep milk was filtered and divided into three portions to obtain different yogurt formulations, as illustrated in Figure 1. The samples were designated as follows: YC—control yogurt (without OP); YOPB—yogurt fortified with 1% OP (*w*/*w*) before pasteurization; YOPA—yogurt fortified with 1% OP (*w*/*w*) after overnight refrigeration at 4 °C.

Each milk portion was poured into sterile 2 L glass beakers. All samples underwent pasteurization at 80 °C for 30 min. In the case of YOPB, freeze-dried olive pomace was added prior to this stage.

After thermal treatment, all samples were cooled to 46 °C and inoculated with 1 mL/L of a freeze-dried starter culture (0.17 DCU/kg of milk equal to 0.031 g/kg of milk, YO-MIX 511 LYO 300 DCU, Danisco, Copenhagen, Denmark). Fermentation was carried out at 44 °C (UN160, Memmert GmbH + Co. KG, Schwabach, Germany), and pH was monitored using a pH meter (420A+, Thermo Orion, Beverly, MA, USA) until a final pH of 4.67 was reached. The pH kinetics of the samples were evaluated by fitting the exponential portion of acidification curves according to Cavallieri and da Cunha [45].

Subsequently, all yogurts were refrigerated at 4 °C for 24 h to stabilize the coagulated gels. After this period, the gels were manually stirred to obtain a stirred yogurt structure. At this point, YOPA was supplemented with 1% (*w*/*w*) freeze-dried olive pomace. All samples (YC, YOPB, and YOPA) were then stirred, transferred into sterile 100 mL polypropylene cups (Roll Srl, Piove di Sacco, Italy), stored at 4 °C, and analyzed after 24 h.

To evaluate the effect of OP powder on yogurt structure formation and its impact on functional properties, the three produced formulations were subjected to the following analyses: water-holding capacity and syneresis, total phenolic content, rheological analysis, and texture analysis. Additionally, acidification curves were monitored.

The optimal formulation, YOPB, was selected for further investigations based on rheological and textural results, which highlighted its superior performance compared to YOPA. Therefore, YOPB was subsequently subjected to further microbiological analysis during the storage period (up to 21 days). In addition, antioxidant activity, surface morphology, color evaluation, and sensory analysis were conducted in comparison with YC.

### 2.9. Analysis of Yogurt Samples

#### 2.9.1. WHC and Syneresis of Yogurt Samples

The water-holding capacity (WHC) of the yogurt samples was determined by centrifuging 25 g of yogurt at 2500 rpm for 20 min at 4 °C (SL1R Plus, Thermo Scientific, Waltham, MA, USA), following the method described by Osorio-Arias et al. [46]. WHC reflects the ability of the yogurt gel network to retain water under centrifugation stress, indicating its structural integrity and resistance to syneresis. WHC was calculated according to the following equation:

$$WHC\left(\%\right)=\left(\frac{Initial\;weight\;of\;yogurt\left(g\right)-Weight\;of\;released\;whey\left(g\right)}{Initial\;weight\;of\;yogurt\left(g\right)}\right)\times 100$$

Syneresis, defined as the spontaneous release of whey from the yogurt gel matrix, was evaluated using the drainage method described by Amatayakul et al. [47]. Briefly, 30 g of yogurt sample was placed on a stainless-steel sieve with a mesh size of 800 µm (Endecotts Ltd., London, UK) and left to drain at room temperature for 15 min. Syneresis was calculated as the percentage of whey separated from the gel in relation to the initial weight of the yogurt using the following formula:

$$Syneresis\left(\%\right)=\left(\frac{Weight\;of\;released\;whey\left(g\right)}{Initial\;weight\;of\;yogurt\left(g\right)}\right)\times 100$$

#### 2.9.2. Total Phenolic Content of Yogurt Samples

Total phenolic content was determined following the extraction method described by Vázquez et al. [48], with slight modifications. A total of 8 g of each yogurt sample was weighed into a 25 mL volumetric flask, and 10 mL of a methanol/water solution (50:50, *v*/*v*; Carlo Erba, Milan, Italy) was added. Subsequently, 0.5 mL each of Carrez I and Carrez II solution (Supelco, Merck Life Science, Milan, Italy) and 5 mL of acetonitrile (Carlo Erba, Milan, Italy) were added. The flask was brought to volume with the same methanol/water solution (50:50, *v*/*v*). After each addition, the mixture was vortexed for 1 min (ZX3, Velp Scientifica, Usmate, Italy). The content was transferred to a 50 mL centrifuge tube, allowed to rest for 25 min at room temperature, and then centrifuged at 7800 rpm for 15 min at 5 °C. The resulting supernatant was collected and transferred to a graduated cylinder for volume measurement.

TPC was quantified following the Folin–Ciocalteu colorimetric method as adapted by Noriega-Rodríguez et al. [34] with some modifications as previously described in Section 2.3. Results were expressed as milligrams of GAE per 100 g of fresh weight (mg GAE/100 g FW).

#### 2.9.3. Rheological Analyses of Yogurt Samples

Rheological properties of the yogurt samples were evaluated using a rheometer (MCR-92, Anton Paar, Graz, Austria) equipped with a parallel plate geometry (diameter: 50 mm; gap: 0.5 mm) at 25 °C according to Yeung et al. [49].

Steady shear tests were performed over a shear rate range of 1–1000 s^−1^ to determine flow behavior parameters, including the flow behavior index (n), consistency index (K), Casson yield stress (σ_ac_), and apparent viscosity at 50 s^−1^.

Frequency sweep tests were conducted to evaluate the viscoelastic properties of the yogurt samples. Storage modulus (G′), loss modulus (G″), loss tangent (tan δ), and complex viscosity (η*) were obtained across a range of angular frequencies (0.63–62.8 rad/s at 2% strain) under the linear viscoelastic region.

#### 2.9.4. Texture Analysis of Yogurt Samples

Textural properties of the yogurt samples were evaluated using a TA.XT plus texture analyzer (Stable Micro Systems, Godalming, UK) equipped with a P/25 aluminum cylindrical probe (25 mm diameter, 11305, Stable Micro System) and a 5 kg load cell. A penetration test was performed to measure firmness, stickiness, work of shear, and work of adhesion.

Yogurt samples were tested directly in polypropylene cups. The probe was driven vertically into the yogurt to a depth of 20 mm at a constant speed of 1 mm/s and then returned to its initial position at the same speed. The maximum force required to break the gel was used as an indicator of firmness (N), while stickiness was defined as the maximum negative force recorded during the withdrawal phase (N). The work of shear (N·s) and the work of adhesion (N·s) were calculated from the respective force–time curves.

### 2.10. Supplementary Analyses on the Selected Formulation YOPB Sample and YC

#### 2.10.1. Microbiological Analysis of YC and YOPB Samples

Microbiological evaluation was conducted on yogurt samples YC and YOPB during refrigerated storage. Shelf-life was monitored over a 21-day period, with analyses performed on days 1, 7, 15, and 21 to assess the viability of the starter cultures.

A total of 10 g of yogurt was sampled on days 1, 7, 15, and 21 and aseptically transferred into stomacher bags containing 90 mL of buffered peptone water (VWR, Milan, Italy). For each batch and time point, three technical replicates were analyzed. Samples were homogenized for 2 min at room temperature using a stomacher (Stomacher^®^ 400 Circulator Lab Blender, Seward Ltd., Worthing, UK). Serial decimal dilutions were prepared, and 0.1 or 0.5 mL aliquots of appropriate dilutions were plated in duplicate on selective media. *Lactobacillus delbrueckii* subsp. *bulgaricus* was enumerated on MRS agar acidified at pH 5.4 (VWR, Milan, Italy), incubated anaerobically at 42.5 °C for 48 h. *Streptococcus thermophilus* was enumerated on STM agar (VWR, Milan, Italy) (per liter: tryptone 10 g, yeast extract 5 g, lactose 10 g, K_2_HPO_4_ 2 g, agar 10 g), incubated aerobically at 37 °C for 24 h [50].

At the end of the storage period (day 21), additional microbiological analyses were performed to detect spoilage organisms and foodborne pathogens. *Listeria monocytogenes, Salmonella* spp., *Pseudomonas* spp., *Enterobacteriaceae*, *Escherichia coli*, presumptive Enterococci, coagulase-positive staphylococci, aerobic spore-forming bacteria, yeasts, and moulds were assessed as previously described in Section 2.7.

#### 2.10.2. Antioxidant Activity of YC and YOPB Samples

The antioxidant capacity of the YC and YOPB yogurt samples was measured using the same extract previously employed for the quantification of TPC on yogurt samples, as reported by Vázquez et al. [48], with slight modifications, as previously described in Section 2.9.2. Subsequently, the AA was assessed by an ABTS assay following the method reported by Dahdah et al. [7], as detailed in Section 2.4. Results were expressed as µmol of Trolox equivalent per gram of fresh weight (µmol TE/g FW).

#### 2.10.3. Scanning Electron Microscopy Analysis of YC and YOPB Samples

The microstructure of the YC and YOPB yogurt samples was examined using SEM with a Zeiss EVO LS10 microscope (Carl Zeiss Microscopy GmbH, Oberkochen, Germany) operated in high-vacuum mode. Sample preparation, including chemical fixation, dehydration, critical point drying, and gold/palladium coating, was performed following the same procedure described for olive pomace in Section 2.5.

#### 2.10.4. Color Attributes of YC and YOPB Samples

The color of the YC and YOPB yogurt samples was evaluated using a Minolta CR-300 colorimeter (Konica Minolta Sensing, Osaka, Japan), calibrated prior to measurement with a standard white calibration tile provided by the manufacturer. Measurements were carried out using a CR-300 measuring head under a D65 illuminant and a standard 10° observer angle, in accordance with CIE guidelines. Color parameters were recorded in the CIE Lab* color space: L*: lightness (L = 0: black, L = 100: white), a*: green-to-red axis (−a* = green, +a* = red), b*: blue-to-yellow axis (−b* = blue, +b* = yellow).

#### 2.10.5. Sensory Evaluation of YC and YOPB Samples

Sensory evaluation was performed at the Sensory Analysis Laboratory of the Department of Agriculture, University of Sassari, through an acceptability test involving 120 consumers aged between 19 and 65 years. Prior to participation, all subjects provided written informed consent. Individuals with lactose intolerance or known allergies to olives or olive-derived products were excluded from the test (*n* = 20). The test was conducted in a single session using a 9-point hedonic scale (1 = “dislike extremely”; 9 = “like extremely”) to evaluate the acceptability of YC and YOPB samples. Yogurts were presented monadically with three-digit random codes in a balanced and randomized order. Panellists were instructed to rinse their palates with water between samples. Participants evaluated the following sensory attributes: texture, flavor, and overall acceptability.

### 2.11. Statistical Analysis

The data were expressed as the mean ± standard deviation, and all experiments were performed in triplicate. Statistical analysis was conducted utilizing the Statgraphics Centurion XVI for Windows software package (version 16.2.04; Statpoint Technologies, Inc., Warrenton, Virginia, VA, USA). A one-way analysis of variance (ANOVA) followed by Tukey’s HSD test (*p* < 0.05) was conducted on the data relating to the differences in acidification curves, water-holding capacity, syneresis, total phenolic content, rheological behavior, texture, antioxidant activity, color, and sensory evaluation of different yogurt formulations. Bacterial count data were log_10_-transformed prior to statistical analysis. The effects of storage time (1, 7, 14, 21 days) and yogurt formulation (YC and YOPB) were evaluated by two-way analysis of variance (ANOVA) using the SPSS Statistics software (version 22, IBM Corp., Armonk, NY, USA). When significant differences were found (*p* < 0.05), mean comparisons were performed using Tukey’s post hoc test.

## 3. Results and Discussion

### 3.1. Proximate Composition, TPC, AA, and Color Properties of OP

The physicochemical characteristics of freeze-dried olive pomace from the Bosana cultivar are summarized in Table 1. Key parameters include moisture content, macronutrient composition (ash, lipids, total dietary fiber), water activity (a_w_), elemental composition (C, H, N), total phenolic content, antioxidant capacity, and color attributes. It is well established that these parameters have a pivotal role in maintaining and/or enhancing rheological and microstructural characteristics of dairy products. Low residual moisture content is a key requirement for stabilizing food powders, as it significantly reduces the risk of microbial proliferation. In this study, the OP powder showed a low moisture level (6.48%) and a water activity of 0.45. These findings are consistent with the safety thresholds reported for dried food products by Zambrano et al. [51], who recommended maintaining a_w_ below 0.60–0.65 and moisture content under 10% to ensure microbiological stability. Lipid content (16.53%) was consistent with the values reported in Arbequina olive pomace from California (USA) by Sinrod et al. [52], while total dietary fiber (63.14%) matched the range described by Dahdah et al. [6] for the same product, highlighting its nutritional relevance. In addition, total dietary fiber is known to cause a reduction in syneresis, causing a thickening and water-binding effect [53]. The ash content of the OP powder was 5.08 ± 0.02 %, reflecting a consistent residual mineral fraction. This value aligns with previous findings in the literature, which report ash contents for olive pomace typically ranging between 4% and 7% depending on the cultivar, the processing method, and the relative amount of stone or woody fraction in the pomace [13,54,55]. The estimated protein content was 6.06%, a value consistent with that previously reported for Bosana cv [6] (6.13%). In comparison, the finding obtained in this study was lower than that observed in Tunisian olive pomace waste (17%) [56] but higher than the Chemlal variety from north-central Algeria (4.25%) [57]. The proximate composition was employed to calculate the digestible carbohydrate fraction by the difference, yielding a result of 9.19%.

TPC was 23.65 ± 1.19 g GAE/kg dry weight (DW), lower than values reported in the literature. Higher concentrations were observed in Bosana pomace (42.67 g GAE/kg DW) [6] and in a blend of Portuguese cultivars (40.9 g GAE/kg DW) [55]. Nunes et al. [58] also reported TPC ranging from 30.5 to 38.3 g GAE/kg DW in freeze-dried samples, while Gómez-Cruz et al. [59] found values between 25.8 and 43.6 g GAE/kg DW, depending on the extraction method applied. In contrast, markedly lower TPC values were reported by Pikuli and Devolli [60] for Kalinjoti pomace (3.75 g GAE/kg DW) and by Martins et al. [61] for exhausted olive pomace (EOP), with values ranging from 2.00 to 4.09 g GAE/kg DW. The observed discrepancies in the published literature may be ascribed to the following factors: variations in olive cultivar, pomace type (fresh, dried, or exhausted), decanter system (two- or three-phase), and extraction protocols [60]. The presence of OP powder in yogurt can represent a significant source of polyphenols, which have been demonstrated to possess health-promoting properties.

The antioxidant capacity (AA), determined by the ABTS assay, was found to be 130.26 ± 3.65 µmol TE/g DW. Martins et al. [61] reported ABTS-based antioxidant capacities ranging from 186.7 to 691.5 µmol TE/g of dry extract, with the highest value achieved using water maceration at 50 °C. Similarly, Gómez-García et al. [62] evaluated the antioxidant capacity of exhausted olive pomace using an orbital incubator and obtained values between 57.1 and 75.6 mg TE/g DW (equivalent to 228.1–302.1 µmol TE/g DW), depending on the extraction solvent, with water yielding the most effective result. As previously observed for total phenolic content, ABTS-derived antioxidant capacity values reported in the literature exhibit considerable variability. This can be attributed to differences in cultivar, drying processes, and extraction methodologies. These methodologies include aqueous maceration, enzyme-assisted, ultrasound-assisted, and mechanically agitated extraction techniques. In the present study, the extracts used to evaluate AA were obtained through solvent extraction employing acetone and methanol. It is hypothesized that this may be the reason for the lower AA in comparison to the literature data. In conclusion, the antioxidant activity of the powder under investigation may confer a number of potential benefits to the yogurt, including the retardation of lipid oxidation and the action against the presence of spoilage or pathogenic microorganisms, thus increasing its shelf-life.

The color attributes of the OP powder were evaluated using the CIE Lab system, yielding values of L* = 37.75 ± 0.44, a* = 5.43 ± 0.15, and b* = 13.62 ± 0.74. The obtained results are in close agreement with those reported by Sinrod et al. [52] for fresh Arbequina olive pomace subjected to different treatments, which exhibited L* values ranging from 38.5 to 52.3 and a* values between 2.07 and 8.57, although slightly higher b* values were observed (21.62–29.04). Similarly, Lammi et al. [50] reported L* values ranging from 29.1 to 65.8, a* values between 5.3 and 9.7, and b* values from 11.6 to 18.6, depending on the treatment applied to the olive pomace. These values are consistent with the typical chromatic profile of olive-derived by-products, which are generally characterized by low lightness, a slight red tint, and moderate yellowness. It is evident that the incorporation of powder will invariably influence the color of the yogurts produced, which must meet the acceptance criteria of consumers.

### 3.2. Microbiological Analysis of OP

No pathogenic microorganisms were detected in the OP, and only a low concentration of aerobic spore-forming bacteria was found (2.86 ± 0.12 log_10_ CFU/g), indicating an overall minimal microbial load. This limited contamination is likely attributable to the freeze-drying process, which effectively reduces water activity and inhibits microbial proliferation, as well as to the intrinsic antimicrobial properties of OP [63]. Previous studies have highlighted the antimicrobial efficacy of OP extracts against a variety of foodborne pathogens. For instance, Friedman et al. [64] demonstrated inhibitory activity of OP powder against *Escherichia coli O157:H7*, *Salmonella enterica*, *Listeria monocytogenes*, and *Staphylococcus aureus*, with particularly strong effects against *S. aureus*. This broad-spectrum antimicrobial activity is primarily attributed to the presence of bioactive compounds, including monounsaturated fatty acids and phenolic constituents such as hydroxytyrosol, tyrosol, oleuropein, and their glycosylated derivatives [65].

The native microbiota of olive pomace is predominantly composed of environmental microorganisms derived from soil and freshwater ecosystems. However, the presence of fecal-associated bacteria has also been reported in the literature, including genera such as *Dialister* and *Prevotella*, as well as members of the *Ruminococcus*–*Eubacterium*–*Clostridium* cluster [66]. Other opportunistic species occasionally found in OP include *Enterobacter cloacae*, *Aeromonas hydrophila*, *Pseudomonas aeruginosa*, and *Serratia odorifera* [67]. Given the potential occurrence of such microorganisms, comprehensive microbiological assessments are crucial to exclude contamination and ensure the safety of OP for food applications. In this context, the observed low microbial load confirms the efficacy of freeze-drying as a preservation method capable of significantly reducing or eliminating microbial contaminants.

### 3.3. Microstructure Analysis of OP

**Figure 2:**
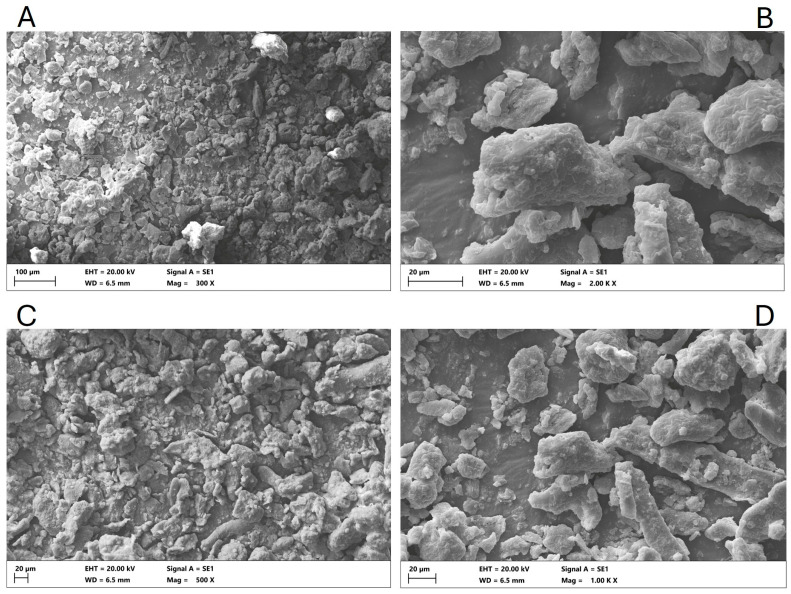
Scanning electron microscopy pictures of OP powder obtained from the Bosana olives, at different scale bars and magnifications (**A**: 100 µm – 300 X; **B**: 20 µm – 2000 X; **C**: 20 µm – 500 X; **D**: 20 µm – 1000 X).

The microstructure of OP powder was examined by scanning electron microscopy, which revealed an irregular and heterogeneous surface morphology, with some regions displaying smoother textures. The OP particles appeared as compact aggregates formed by fractured cell wall fragments, interspersed with voids and porous zones (Figure 2). The analysis of particle size revealed a heterogeneous distribution, with a mean equivalent circular diameter of 5.0 µm and values ranging from 0.67 to 141.48 µm. The majority of particles were observed to be within the 1–5 µm range (approximately 54%), with a subsequent prevalence of particles smaller than 1 µm (approximately 24%) and particles measuring 5–10 µm (approximately 12%). A negligible proportion of particles exceeded 10 µm: approximately 9% were in the 10–50 µm range, and less than 1% were above 50 µm. This distribution indicates the prevalence of fine particles, interspersed with sporadic larger fragments, which is consistent with the irregular structure of the olive pomace matrix.

These aggregates exhibited a characteristic stone-like appearance, attributed to the presence of waxes, lipid compounds, and a high content of total dietary fiber, particularly lignin and cellulose [68]. This heterogeneity reflects the complex structure of the olive pomace matrix, in agreement with previous SEM analyses of olive pomace matrices [69,70,71]. Moreover, the freeze-drying process likely contributed to preserving the porous and fibrous architecture by preventing cell matrix collapse and minimizing thermal degradation.

### 3.4. Effect of OP on Yogurt Characteristics

#### 3.4.1. Acidification Curves of Yogurt Formulations

The acidification kinetics of the different yogurt formulations were evaluated by monitoring the pH decrease over time during fermentation. It should be noted that YC and YOPA were identical during the fermentation phase, as olive pomace was added to YOPA only after the overnight refrigeration phase at 4 °C. No significant differences were observed in the time required to reach the target pH of 4.67 among treatments, with all formulations showing comparable acidification trends (*p* > 0.05). The time required to reach pH 4.67 was on average 5 h 14 min for YC, 5 h 05 min for YOPA, and 4 h 53 min for YOPB (Table 2). The initial pH (measured at time 0, after inoculation with starter cultures) differed significantly among treatments (*p* < 0.05), with YOPB exhibiting a slightly lower value (6.41) compared to YC (6.53) and YOPA (6.51). This initial difference may be attributed to the intrinsic acidity of OP, which contains considerable amounts of phenolic compounds—such as hydroxytyrosol, tyrosol, and oleuropein—known to contribute to mild acidity in food matrices [7,9,72]. Despite this initial difference, no statistically significant differences were observed in the process rate, *k*, among the treatments (*p* > 0.05). The process rates were 8.40 ± 0.42 for YC, 8.95 ± 0.49 for YOPA, and 7.75 ± 0.21 for YOPB, indicating that the presence of OP did not have a substantial effect on fermentation kinetics.

These findings are consistent with previous studies showing that the incorporation of olive-derived ingredients into dairy matrices—such as olive leaf extract [73,74,75,76] or olive leaf powder [77]—does not negatively affect the growth and viability of lactic acid bacteria or the overall fermentation process. Notably, Barukčić et al. [73] observed that although the addition of olive leaf extract led to a faster decrease in pH and shorter fermentation time, it did not compromise the viability of the yogurt starter culture.

In conclusion, although the addition of OP prior to pasteurization slightly lowered the initial pH of the milk, it did not influence the acidification kinetics. These results confirm the feasibility of incorporating OP into milk–yogurt formulations as a functional ingredient without adversely affecting fermentation performance or processing time [76].

#### 3.4.2. Impact of OP Addition on the WHC and Syneresis of Yogurt Samples

**Figure 3:**
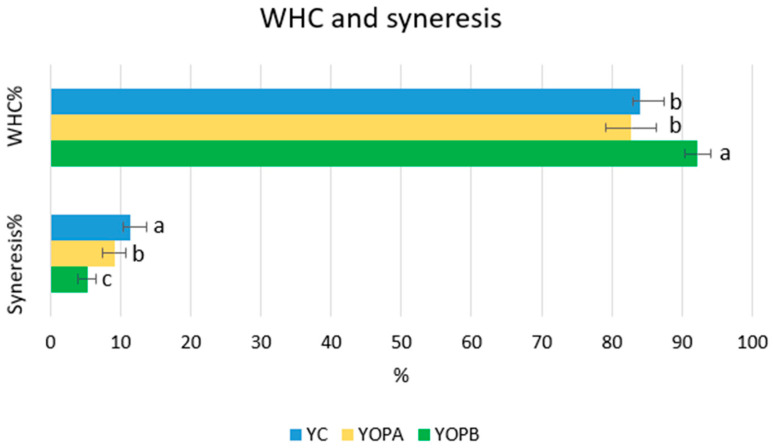
WHC and syneresis values of the yogurt samples. Values are presented as mean ± standard deviation (*n* = 3). Different superscripts within each column indicate significant differences following one-way analysis of variance (ANOVA) and Tukey’s HSD test (*p* < 0.05).

Water-holding capacity is a fundamental quality attribute of yogurt, as it reflects the capacity of the protein gel network to retain water. This attribute is closely related to product texture, stability, and consumer acceptability. In the present study, the addition of olive pomace prior to pasteurization significantly enhanced the WHC of sheep milk yogurt, reaching 92.21 ± 1.88%, compared to 84.02 ± 3.33% for YC and 82.75 ± 3.56% for YOPA (*p* < 0.05) (Figure 3). This finding suggests that incorporating OP before thermal treatment may promote stronger interactions between OP-derived phenolic compounds and dietary fibers, as well as milk proteins—particularly relevant in sheep milk, which is naturally rich in proteins and is renowned for its remarkable water retention capacity [75,78]. These interactions may contribute to the enhanced structural integrity of the gel matrix, leading to a more stable protein network capable of retaining water under mechanical stress, such as centrifugation. This result is consistent with previous studies investigating the fortification of yogurt with olive-derived ingredients. The incorporation of olive leaf extract into cow, goat, and sheep milk yogurts led to a significant improvement in water-holding capacity across all formulations, with sheep milk yogurt displaying the greatest enhancement. These findings were reported by Tarchi et al. [75], who attributed the improved WHC to protein–polyphenol interactions, facilitated by the distinct compositional and structural properties of each milk type. It is well established that during fermentation, milk proteins form a gel matrix capable of entrapping water molecules, thus contributing to yogurt’s water retention capacity. Similarly, Barukčić et al. [73] observed higher WHC values in yogurt enriched with olive leaf extract, with the effect persisting until day 28 of storage, although no differences were found among the different concentrations used. Conversely, Aydın et al. [76] did not detect significant differences in WHC between control samples and those fortified with olive leaf extract during the entire storage period.

The evaluation of syneresis, defined as the spontaneous separation of whey from the yogurt gel matrix, revealed significant differences among the formulations (*p* < 0.05) (Figure 3). YC exhibited the highest syneresis value (11.32 ± 2.41%), indicating a lower water retention capacity. YOPA showed an intermediate value (9.12 ± 1.64%), while YOPB demonstrated the lowest syneresis (5.27 ± 1.26%).

These findings confirm that incorporating OP before thermal treatment can enhance the water retention capacity of the yogurt matrix, likely due to the formation of a more compact and stable protein–polyphenol network that limits whey separation under gravitational stress. The intermediate value observed in YOPA further indicates that the timing of OP addition plays a key role in modulating the physical stability of the gel, with pre-pasteurization incorporation being more effective.

These results are consistent with previous studies [79,80], which reported a significant reduction in syneresis following the addition of encapsulated olive leaf extracts. In those studies, the improved stability was attributed to an increase in total solids content, which enhanced the gel structure and reduced serum release. Similar findings have been reported in yogurt enriched with other fiber-rich ingredients, such as orange pomace powder, where increased WHC and reduced syneresis were observed—likely due to the water-binding capacity of dietary fibers and their ability to reinforce the protein network [81]. Conversely, Aydın et al. [76] reported no significant differences in syneresis between fortified and control yogurts, highlighting that the impact of olive-derived ingredients may vary depending on formulation parameters, including fiber content, dosage, and processing conditions.

#### 3.4.3. Effect of OP Fortification on Total Phenolic Content of Yogurts

As expected, the incorporation of olive pomace into the yogurt matrix significantly enhanced its total phenolic content (*p* < 0.05). As expected, YC exhibited the lowest TPC value (2.54 ± 0.46 mg GAE/100 g FW), whereas both enriched formulations with OP (YOPA and YOPB) revealed statistically higher values (15.67 ± 0.90 and 15.30 ± 1.97 mg GAE/100 g FW, respectively). No significant variation was observed between YOPA and YOPB. These results indicate that the addition of OP, regardless of the stage of incorporation, induced an approximately six-fold increase in TPC compared to the control, confirming the efficacy of both methods in enhancing the polyphenolic profile of the yogurt.

Ribeiro et al. [24] reported higher TPC values than those obtained in the present study. However, these values were obtained through the fortification of yogurt derived from whole cow’s milk (supplemented with 3% milk powder (*w*/*v*)) with liquid-enriched (LOPP) olive pomace powders (30.69 mg GAE/100 g FW). Slightly higher values were observed for the pulp-enriched (POPP) sample (19.15 mg GAE/100 g FW) and the control (5.81 mg GAE/100 g FW). The fortifications were conducted with 2% of POPP and LOPP, and the TPC content of raw matrices was found to be 848.48 and 496.59 mg/100 DW, respectively. Additionally, other studies employing olive leaf extracts [75,79,82] demonstrated similar trends, with TPC increasing proportionally to the amount of phenolic-rich extract used. In conclusion, the incorporation of a natural olive oil by-product has been demonstrated to enhance the health-promoting potential and the nutritional value of yogurt.

#### 3.4.4. Modification of the Rheological Behavior of Yogurt by OP Addition

The rheological behavior of the yogurt samples was evaluated through both steady shear and frequency sweep test, revealing significant differences among formulations.

According to the steady shear analysis, all yogurt samples exhibited non-Newtonian shear-thinning behavior, as evidenced by flow behavior indices (n) lower than 1 across all formulations (Table 3). The steady shear analysis revealed that YC exhibited the most pronounced shear-thinning behavior (n = −0.92), followed by YOPB (−0.85) and YOPA (−0.81). The values were found to be significantly different (*p* < 0.05), thus indicating that the addition of olive pomace, led to a slight reduction in the pseudoplastic nature of the yogurt matrix.

With regard to the consistency index (K), YOPB and YC showed the highest values (16.32 and 15.86 Pa·sⁿ, respectively), with YOPA exhibiting a significantly lower value (10.74 Pa·sⁿ; *p* < 0.05). This finding suggests that viscous nature of yogurt was maintained or even augmented in YOPB, a phenomenon that may be attributable to the interactions between the fiber components of OP, incorporated prior to the pasteurization stage, and the protein network.

The apparent viscosity at 50 s^−1^ followed a comparable trend, with YOPB showing the highest value (0.67 Pa·s; *p* < 0.05), significantly higher than both YOPA and YC. This result may suggest a greater ability of YOPB to preserve its consistency under moderate-shear conditions, which could be relevant during processing or consumption.

The Casson yield stress (σ_ac_), representing the initial stress needed to initiate flow, was significantly highest in YC (1.90 Pa), followed by YOPB and YOPA (*p* < 0.05), suggesting a reduced resistance to flow upon pomace addition.

With reference to the frequency sweep measurement obtained at 6.28 rad/s (Table 4), it can be observed that the control yogurt displayed significantly higher values of G′ and G″ (324.37 and 93.83 Pa, respectively), followed by YOPB and YOPA, indicating that pomace addition decreased both the elastic and viscous components of the gel matrix, as already observed in the study by Yeung et al. [49] after addition of pectic polysaccharides, confirming the weak gel nature of the yogurt matrix.

Complex viscosity (η*) mirrored this pattern, with YC maintaining the highest value (46.10 Pa·s) and YOPA the lowest (29.55 Pa·s). No significant differences were observed in tan δ among samples (*p* > 0.05), indicating that the predominance of the elastic component was consistently maintained across treatments.

As demonstrated by the present results, YOPB exhibited rheological behavior that was largely comparable to the control and often preferable to YOPA.

To the best of our knowledge, this is the first study to evaluate in detail the rheological behavior—both under steady and dynamic shear—of yogurts enriched with olive pomace.

#### 3.4.5. Influence of OP Incorporation on the Textural Properties of Yogurt Samples

The textural attributes of the yogurt samples, including firmness, work of shear, work of adhesion, and stickiness, are summarized in Table 5. The incorporation of OP significantly influenced selected texture parameters, with the timing of addition—either before pasteurization or after the overnight refrigeration process—emerging as a critical factor.

Firmness, defined as the maximum force required to achieve a specific deformation [83], did not differ significantly between the control (YC: 0.43 ± 0.03 N) and YOPB (0.44 ± 0.04 N). However, YOPA exhibited significantly lower firmness (0.39 ± 0.04 N; *p* < 0.05), suggesting a weaker protein gel structure when OP was added post-fermentation.

The work of shear (N·s), defined as the energy required to deform the yogurt matrix under shear [84], mirrored the firmness trends. YOPA (5.48 ± 0.54 N·s) was significantly lower (*p* < 0.05) than YC (6.12 ± 0.46 N·s) and YOPB (6.21 ± 0.52 N·s), indicating a less resilient gel structure.

The work of adhesion (N·s), which represents the energy required to detach the probe from the yogurt sample after compression [84], differed significantly among the formulations (*p* < 0.05). YOPA exhibited significantly higher (less negative) values (−1.96 ± 0.47 N·s) compared to YC (−2.97 ± 0.50 N·s) and YOPB (−3.23 ± 0.74 N·s), suggesting a lower degree of adhesive strength when OP was added after fermentation.

Stickiness, measured as the peak negative force during probe withdrawal [85], was also significantly higher in YOPA (−0.12 ± 0.02 N) compared to YC (−0.15 ± 0.02 N) and YOPB (−0.16 ± 0.02 N) (*p* < 0.05), further confirming a less structured and adhesive gel.

The distinct textural behaviors observed between YOPB and YOPA can be attributed to the timing of OP addition in relation to thermal processing. When OP was incorporated into the milk prior to pasteurization, it is hypothesized that the phenolic compounds and fibers present in the OP interacted with the milk proteins during the process of heat-induced denaturation. The aforementioned conditions are favorable for the formation of stable protein–polyphenol complexes, which contribute to the development of a more cohesive gel network with improved mechanical strength and water-holding capacity [75].

In contrast, the addition of OP after overnight refrigeration occurred within a fully formed yogurt matrix at 4 °C and pH 4.50—conditions that may limit the potential for effective protein–polyphenol interactions [86]. This is likely to have resulted in a looser, less cohesive gel network. It is widely accepted that heat-induced protein denaturation is a key factor in facilitating the formation of stable protein–polyphenol complexes in dairy systems, including ovine yogurt.

In summary, the timing of OP incorporation plays a key role: early addition promotes structural reinforcement, whereas post-fermentation supplementation may compromise the mechanical integrity of the yogurt matrix. It is noteworthy that YOPB exhibited results that were comparable to the control and superior to those of YOPA in all measured parameters.

In consideration of the results obtained, with particular reference to rheological behavior and textural properties, YOPB was identified as the most suitable formulation. Consequently, it was selected for further analyses, including microbiological characterization, antioxidant activity, microstructural observation, color evaluation, and sensory analysis. All analyses were conducted concurrently with the YC sample.

### 3.5. Microbiological Characterization of YC and YOPB Yogurt Samples

Viable cell counts are a fundamental parameter in the development and quality assessment of fermented dairy products, serving as a key indicator of microbial viability and functionality. The use of high-quality raw milk supports consistent fermentation performance and minimizes the risk of interference from residues [87]. It is generally accepted that yogurt should contain at least 10^7^ CFU/mL of viable cells, expressed as the combined counts of the typical starter cultures, namely *Streptococcus thermophilus* and *Lactobacillus delbrueckii* subsp. *bulgaricus*, to ensure its functional and sensory attributes [88,89].

**Figure 4:**
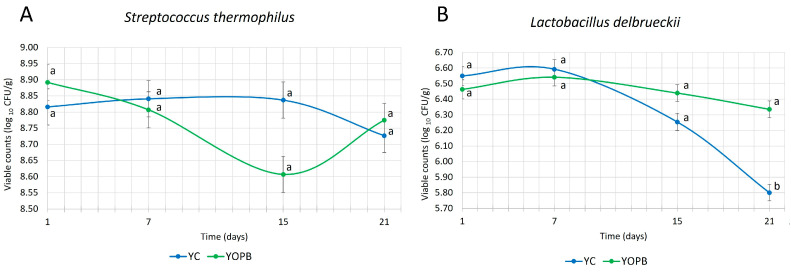
Viable counts of *Streptococcus thermophilus* (**A**) and *Lactobacillus delbrueckii* subsp. *bulgaricus* (**B**) of YC and YOPB yogurt samples during storage at 4 °C over 21 days. Data are expressed as mean log_10_ CFU/g ± standard deviation (*n* = 3). Different letters at the same sampling time indicate statistically significant differences (*p* < 0.05).

In the present study, *Streptococcus thermophilus* reached mean values of approximately 8.8 log_10_ CFU/g in both the YC and YOPB samples, with no significant differences observed between the two formulations throughout storage (Figure 4A). Conversely, *Lactobacillus delbrueckii* subsp. *bulgaricus* exhibited lower counts—approximately 2 log units below *S. thermophilus*—and was significantly affected by the presence of OP (*p* < 0.05) (Figure 4B). At both day 1 and day 7 of storage, higher *L. delbrueckii* subsp. *bulgaricus* counts were observed in YC compared to YOPB. Interestingly, while the population remained relatively stable in YOPB throughout storage, a decreasing trend was detected in YC, resulting in an overall reduction of approximately one log unit by the end of the shelf-life.

Comparable findings were reported by Asensio-Vegas et al. [90], who observed variability in *L. delbrueckii* subsp. *bulgaricus* counts in ovine yogurt depending on the commercial starter cultures used. Their results ranged from 4.93 to 7.43 log_10_ CFU/g after one day of storage and from 4.22 to 7.86 log_10_ CFU/g after 28 days. The relatively low initial *L. delbrueckii* subsp. *bulgaricus* counts observed in our study may be attributed to the limited adaptation or suboptimal metabolic activity of the commercial culture in ovine milk.

Although Peker and Arslan [91] reported a growth-promoting effect of olive leaf extract on *S. thermophilus* in low-fat apricot yogurt, no such effect was evident in the YOPB formulation under investigation. However, Servili et al. [92] observed a higher viability of *S. thermophilus* compared to *L. delbrueckii* subsp. *bulgaricus* in a functional milk beverage enriched with phenolic compounds from olive vegetation water, with the latter showing a reduction of approximately 3 log_10_ CFU/mL after 30 days. The observed difference in *L. delbrueckii* counts does not appear to have affected the acidification kinetics, which, as previously reported in Section 3.4.1, showed no significant variation between YOPB and YC. This suggests that the metabolic activity of the starter culture was sufficient to ensure proper fermentation, with no detrimental effects on the technological performance or quality attributes of the yogurt.

The absence of pathogens or spoilage microorganisms in the YC and YOPB samples confirms the microbiological stability and safety of the products. This finding indicates that the incorporation of OP under the experimental conditions used did not adversely affect their shelf-life or overall quality.

### 3.6. Antioxidant Activity of YC and YOPB Yogurt Samples

As expected, YOPB exhibited a markedly higher antioxidant capacity (1.75 ± 0.14 µmol TE/g FW) compared to YC, which showed a significantly lower value (0.55 ± 0.01 µmol TE/g FW) (*p* < 0.05). This corresponds to more than a threefold increase (+218%) in antioxidant activity following the incorporation of olive pomace prior to pasteurization.

This enhancement is likely attributable to the phenolic compounds naturally present in olive pomace, which are well known for their free radical scavenging activity. These findings are consistent with the significantly higher TPC previously observed in YOPB. Ribeiro et al. [24] observed a significant increase in antioxidant activity in cow’s milk yogurts enriched with different types of olive pomace powders, as measured by an ABTS assay. However, these results were lower than those obtained in the present study. In their study, yogurts fortified with LOPP and POPP exhibited antioxidant capacities of 1.24 and 0.69 µmol TE/g FW, respectively, in comparison to 0.15 µmol TE/g FW in the control. These values corresponded to approximately 2.2-fold and 1.2-fold increases over the control. These findings provide further confirmation of the functional role of olive-derived phenolic compounds in enhancing the antioxidant potential of yogurt.

### 3.7. Microstructure of YC and YOPB Yogurt Samples

**Figure 5:**
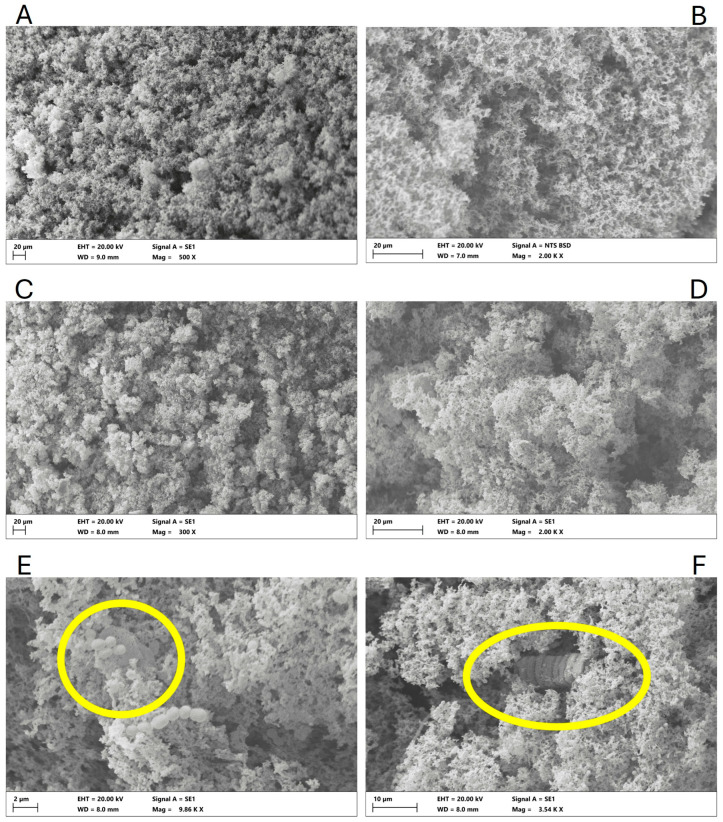
Scanning electron microscopy pictures of experimental yogurts, at different scale bars and magnifications (YC: **A**: 20 µm – 500 X; **B**: 20 µm – 2000 X. YOPB: **C**: 20 µm – 500 X; **D**: 20 µm – 2000 X; **E**: 2 µm – 9860 X; **F**: 10 µm – 3540 X). To assist the reader, the stone-like structures of plant origin presented in Figure 5 (**E**,**F**) are indicated by the use of a circle.

Scanning electron microscopy was employed to examine the microstructure of YC and YOPB at comparable magnifications. YC exhibited the typical morphology of stirred yogurt, with a smooth, continuous protein matrix interspersed with fat globules and microvoids (Figure 5A,B). The network was homogeneous and free from embedded particles, reflecting the absence of added solids.

The YOPB samples exhibited distinctive microstructural features. The incorporation of OP powder did not compromise the overall integrity of the protein network (Figure 5C,D). At higher magnification, compact and irregularly shaped particles of vegetal origin were clearly visible embedded within the protein matrix (Figure 5E,F). These stone-like structures, previously described in Section 3.3, appeared to be distributed throughout the gel in a homogeneous manner, indicating effective integration without disrupting the continuity of the protein network.

These findings provide to support the hypothesis that, when added prior to pasteurization, OP particles become embedded within the protein gel during the process of heat-induced gelation. This integration may contribute to enhanced water retention and gel stability without adversely affecting the texture. To the best of our knowledge, this is the first study to utilize SEM to visualize the incorporation of olive pomace particles into the yogurt matrix, offering novel insights into the microstructural effects of OP fortification.

### 3.8. Color Characteristics of YC and YOPB Yogurt Samples

The color characteristics of the yogurt samples (YC and YOPB) were evaluated using the CIE Lab* color space, with all parameters differing significantly (*p* < 0.05) (Table 6). YC exhibited a light appearance (L* = 65.32 ± 0.45) with a subtle greenish-yellow tint, as indicated by its negative a* value (–3.42 ± 0.03) and positive b* value (6.05 ± 0.13). The chroma (C* = 6.94 ± 0.11) reflected a low-intensity, unsaturated color, while the hue angle (h° = 119.43 ± 0.65) further confirmed a slight inclination towards yellow-green tones.

In contrast, the YOPB sample exhibited a significantly altered color profile. The L* value showed a significant decrease (58.29 ± 0.19), indicative of a darker appearance, while the b* value demonstrated an increase (7.58 ± 0.15), reflecting augmented yellowness. The a* value underwent a shift towards a more neutral tone (–0.42 ± 0.03), thereby suggesting a reduction in the green component. The higher chroma (C* = 7.59 ± 0.15) indicated greater color saturation, and the lower hue angle (h° = 93.13 ± 0.21) signified a shift towards a warmer, more yellow hue.

These color shifts can be attributed directly to the inherent chromatic characteristics of the OP powder (L* = 37.75 ± 0.44, a* = 5.43 ± 0.15, b* = 13.62 ± 0.74). When incorporated into the yogurt matrix, these chromatic characteristics imparted a darker tone and intensified yellow coloration.

These modifications are likely attributable to the presence of phenolic compounds or natural pigments in OP, as hypothesized by Tarchi et al. [75] and previously reported by Barukčić et al. [73] in yogurt enriched with olive leaf extracts.

In further support of these observations, Tarchi et al. [75] reported a reduction in lightness (L*) and an increase in a* and b* values following the addition of olive leaf extracts to yogurt, with effects proportional to the concentration used. Similarly, Aydın et al. [76] observed that the fortification of milk with olive leaf extract led to a reduction in the L* and a* values and an increase in the b* values, resulting in a final product that was greener yet more yellow in comparison to the control.

The total color difference (ΔE*) between YC and YOPB was calculated to be 7.8. According to Salman et al. [93], values that exceed the perceptibility threshold (ΔE* > 3) are visually detectable to the human eye. Thus, the incorporation of OP produced a distinctly perceptible color change in the final yogurt.

### 3.9. Sensory Evaluation of YC and YOPB Yogurt Samples

**Figure 6:**
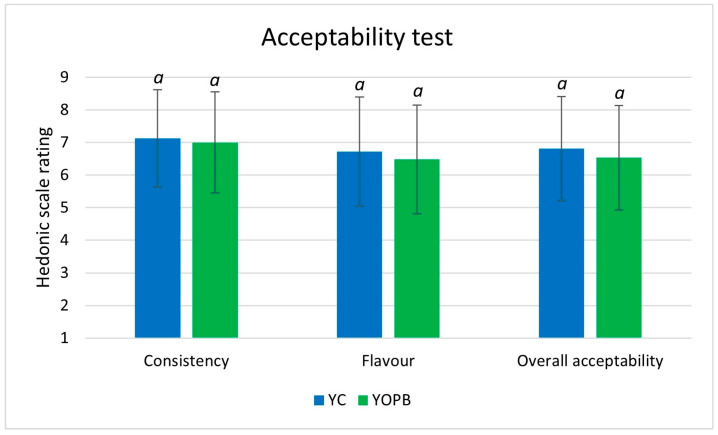
Acceptability test of YC and YOPB samples. Acceptability scores represent the mean ± standard deviation (*n* = 100). Different superscripts within each column indicate significant differences following one-way analysis of variance (ANOVA) (*p* < 0.05).

The results of the acceptability test showed no statistically significant differences (*p* > 0.05) between the YC and YOPB samples in any of the evaluated attributes, including consistency, flavor, and overall acceptability (Figure 6).

These results for consistency show a comparable trend in the instrumental texture analysis, which likewise revealed no significant differences between the two formulations in terms of firmness, work of shear, work of adhesion, and stickiness.

The absence of perceptible differences in sensory attributes is a favorable outcome, indicating that the addition of olive pomace—a by-product of the olive oil industry—prior to pasteurization does not negatively affect the sensory quality of sheep milk yogurt. Both formulations were perceived as equally acceptable by consumers, supporting the feasibility of incorporating OP into yogurt without compromising consumer satisfaction or market potential.

In contrast, several studies on yogurt enriched with olive leaf extracts (OLEs) have reported reduced consumer acceptability at higher inclusion levels due to undesirable changes in flavor, color, and texture. These formulations were often described by panellists as astringent, herbal, or bitter, with higher acceptability observed only in samples containing lower concentrations of OLEs [73,80].

## 4. Conclusions

This study demonstrates the benefits of incorporating freeze-dried olive pomace into sheep milk yogurt as a strategy to enhance its physicochemical, rheological, and textural properties. The incorporation of OP, particularly prior to pasteurization (YOPB), led to substantial improvements in water-holding capacity, reduced syneresis, and increased total phenolic content and antioxidant activity in comparison with the control sample. In addition, the rheological and textural properties exhibited comparable or enhanced characteristics compared to those of the control yogurt. Microbiological analysis confirmed the safety of OP and showed no unfavorable effects on starter culture viability. Notably, YOPB maintained comparable sensory acceptability to the control, confirming consumer acceptance of the fortified product. Furthermore, SEM imaging provided novel evidence of the successful integration of OP into the yogurt matrix, suggesting a positive effect on WHC for YOPB. These results support the use of OP as a sustainable, functional ingredient for dairy applications, promoting circular economy principles and valorizing olive oil by-products.

## Figures and Tables

**Table 1:** Physicochemical, functional, and color properties of freeze-dried olive pomace.

Parameters	^a^ Values
Moisture (g/100 g)	6.48 ± 0.26
Ash (g/100 g DW)	5.08 ± 0.02
Lipids (g/100 g DW)	16.53 ± 0.66
C (%)	55.39 ± 1.58
H (%)	7.56 ± 0.21
N (%)	0.97 ± 0.02
^†^ Estimated protein (%)	6.06 ± 0.13
^‡^ Estimated digestible carbohydrates (%)	9.19
a_w_ (%)	0.45 ± 0.00
Total Dietary Fiber (g/100 g DW)	63.14 ± 0.39
TPC (g GAE/kg DW)	23.65 ± 1.19
ABTS (µmol TE/g DW)	130.26 ± 3.65
L*	37.75 ± 0.44
a*	5.43 ± 0.15
b*	13.62 ± 0.74

^a^ Values are presented as the mean ± standard deviation (n = X). ^†^ Estimated protein (%) as N × 6.25. ^‡^ Estimated digestible carbohydrates (%) by difference: 100−(protein + lipids + total dietary fiber + ash).

**Table 2:** Effect of olive pomace incorporation on initial pH, time to reach pH 4.67, and *k* of yogurt samples.

Sample	Initial pH	Time pH 4.67 (h, min)	*k*_pH_ x (10^−2^) (h^−1^)	R^2^
YC	6.53 ^a^ ± 0.02	5 h 14 ^a^ min ± 39 min	8.40 ^a^ ± 0.42	0.94
YOPA	6.51 ^a^ ± 0.03	5 h 05 ^a^ min ± 31 min	8.95 ^a^ ± 0.49	0.96
YOPB	6.41 ^b^ ± 0.01	4 h 53 ^a^ min ± 37 min	7.75 ^a^ ± 0.21	0.93

Values are presented as the mean ± standard deviation (*n* = 3). Different superscripts within each column indicate significant differences following one-way analysis of variance (ANOVA) and Tukey’s HSD test (*p* < 0.05).

**Table 3:** Steady-shear rheological properties of yogurt samples.

Sample	Flow Behavior Index (n)	Consistency Index (K) (Pa·sⁿ)	Apparent Viscosity ηₐ, _50_ (Pa·s)	Casson Yield Stress σ_ac_ (Pa)
YC	−0.92 ^a^ ± 0.03	15.86 ^a^ ± 2.40	0.48 ^c^ ± 0.02	1.90 ^a^ ± 0.03
YOPA	−0.81 ^b^ ± 0.03	10.74 ^b^ ± 2.48	0.53 ^b^ ± 0.05	1.77 ^c^ ± 0.05
YOPB	−0.85 ^c^ ± 0.01	16.31 ^a^ ± 1.22	0.67 ^a^ ± 0.02	1.84 ^b^ ± 0.01

Values are presented as mean ± standard deviation (*n* = 3). Different superscripts within each column indicate significant differences following one-way analysis of variance (ANOVA) and Tukey’s HSD test (*p* < 0.05).

**Table 4:** Frequency sweep data of yogurt samples.

Sample	Storage Modulus (G′)	Loss Modulus (G″)	Complex Viscosity η*	Tan δ
YC	324.37 ^a^ ± 59.80	93.83 ^a^ ± 16.93	46.10 ^a^ ± 8.44	0.29 ^a^ ± 0.02
YOPA	207.99 ^b^ ± 32.95	58.83 ^b^ ± 19.15	29.55 ^b^ ± 4.90	0.28 ^a^ ± 0.06
YOPB	251.46 ^b^ ± 34.36	79.33 ^a^ ± 11.32	35.24 ^b^ ± 3.57	0.32 ^a^ ± 0.04

Values are presented as mean ± standard deviation (*n* = 3). Different superscripts within each column indicate significant differences following one-way analysis of variance (ANOVA) and Tukey’s HSD test (*p* < 0.05).

**Table 5:** TPA parameters of yogurt samples.

Sample	Firmness (N)	Work of Shear (N·s)	Work of Adhesion (N·s)	Stickiness (N)
YC	0.43 ^a^ ± 0.03	6.12 ^a^ ± 0.46	−2.97 ^b^ ± 0.50	−0.15 ^b^ ± 0.02
YOPA	0.39 ^b^ ± 0.04	5.48 ^b^ ± 0.54	−1.96 ^a^ ± 0.47	−0.12 ^a^ ± 0.02
YOPB	0.44 ^a^ ± 0.04	6.21 ^a^ ± 0.52	−3.23 ^b^ ± 0.74	−0.16 ^b^ ± 0.02

Values are presented as the mean ± standard deviation (*n* = 3). Different superscripts within each column indicate significant differences following one-way analysis of variance (ANOVA) and Tukey’s HSD test (*p* < 0.05).

**Table 6:** Color parameters of YC and YOPB yogurts.

Sample	L*	a*	b*	C	h°
YC	65.32 ^a^ ± 0.45	−3.42 ^b^ ± 0.03	6.05 ^b^ ± 0.13	6.94 ^b^ ± 0.11	119.43 ^a^ ± 0.65
YOPB	58.29 ^b^ ± 0.19	−0.42 ^a^ ± 0.03	7.58 ^a^ ± 0.15	7.59 ^a^ ± 0.15	93.13 ^b^ ± 0.21

Values are presented as mean ± standard deviation (*n* = 3). Different superscripts within each column indicate significant differences following one-way analysis of variance (ANOVA) and Tukey’s HSD test (*p* < 0.05).

## Data Availability

The original contributions presented in this study are included in the article. Further inquiries can be directed to the corresponding author.
